# P-463. The Return of Mycoplasma pneumoniae: An ‘Atypical’ 2024-2025 Season at a Community Children’s Hospital

**DOI:** 10.1093/ofid/ofaf695.678

**Published:** 2026-01-11

**Authors:** Stanley Toyberman, Tibisay Villalobos

**Affiliations:** Lehigh Valley Reilly Children's Hospital, Allentown, Pennsylvania; Lehigh Valley Reilly Children's Hospital, Allentown, Pennsylvania

## Abstract

**Background:**

*Mycoplasma pneumoniae* is a common cause of respiratory illness in children. During the COVID-19 pandemic, *M. pneumoniae* infections decreased globally. In the Fall of 2023, various countries identified a reemergence of this bacterium. At Lehigh Valley Health Network (LVHN), the emergency departments and the children’s hospital experienced an increase of Mycoplasma infections starting in late Spring of 2024. The aim of this study was to compare the percentage of positive *M. pneumoniae* PCR results to determine if there was a significant local increase in the number of Mycoplasma infections over the past year.

**Figure:**
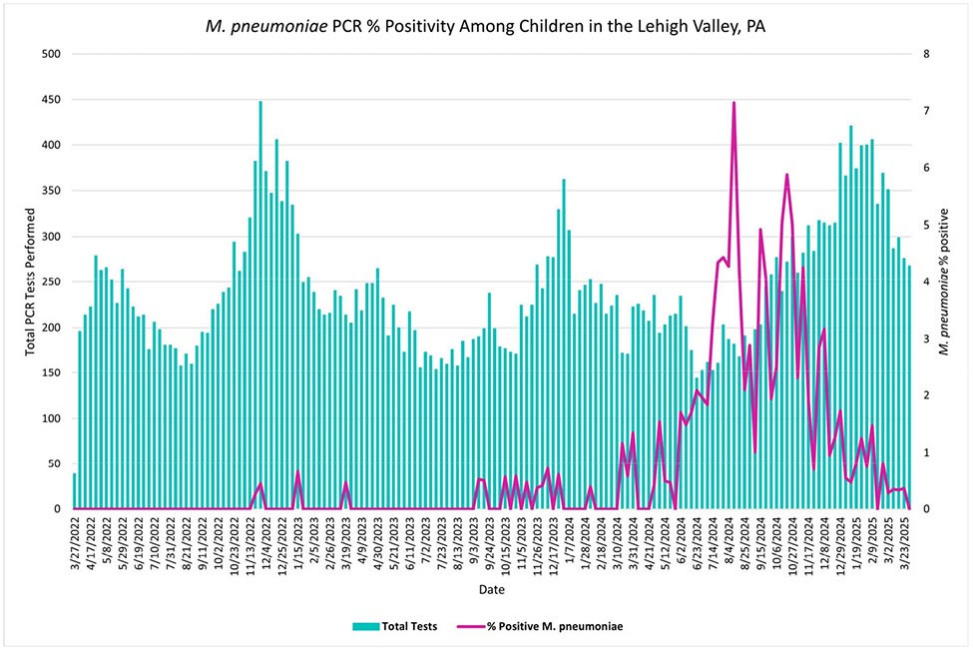

Figure showing the total number of PCR multiplex tests performed (left axis) and the percentage of positive Mycoplasma pneumoniae PCR tests (right axis) from March 2022 through March 2025.

**Methods:**

A retrospective review of *M. pneumoniae* PCR tests from the BIOFIRE RP2.1™ Panel (BioFire Diagnostics, Salt Lake City, Utah) was performed in patients younger than 18 years who had testing performed at LVHN emergency departments and inpatient pediatric units. Available test results were reviewed starting from March 2022, when the institution began using the multiplex panel that included *M. pneumoniae*, through March 2025.

**Results:**

During 2022 and 2023, the weekly *M. pneumoniae* PCR positivity remained below 1%. The weekly PCR positivity rate started increasing in Spring 2024 and reached a peak of 7.1% in August 2024 despite a lower number of tests performed (182 tests). The percentage of positive PCR tests remained elevated until late October 2024, with a PCR positivity of 5.9%. From March 31, 2024 to March 29, 2025, 250 children tested positive for *M. pneumoniae*. This is compared to only 15 positive PCR tests during the same time period the year prior and 6 positive tests the year before that. The mean weekly positivity rate between March 31, 2024 and March 29, 2025 was 1.98 compared to 0.13 the prior year (p < 0.0001).

**Conclusion:**

Following a period of minimal *M. pneumoniae* activity after the COVID-19 pandemic, the percentage of *M. pneumoniae* positive PCR test results significantly increased in our community over the last year. During a time with low testing and low circulation of other respiratory viruses, there was a notable peak of Mycoplasma infection during the Summer of 2024 which continued into the late Fall. Further monitoring is needed to evaluate the periodicity of Mycoplasma infection in the post-pandemic period.

**Disclosures:**

All Authors: No reported disclosures

